# Identification and characterization of miRNAs in the gills of the mud crab (*Scylla paramamosain*) in response to a sudden drop in salinity

**DOI:** 10.1186/s12864-018-4981-6

**Published:** 2018-08-14

**Authors:** Huan Wang, Hongling Wei, Lei Tang, Junkai Lu, Changkao Mu, Chunlin Wang

**Affiliations:** 1 School of Marine Science, Ningbo University, Ningbo, 315211 Zhejiang China; 2 Key Laboratory of Applied Marine Biotechnology, Ministry of Education, Ningbo University, Ningbo, 315211 Zhejiang China

**Keywords:** KEGG pathway, MicroRNA, *Scylla paramamosain*, Sudden drop in salinity

## Abstract

**Background:**

The mud crab (*Scylla paramamosain*) is a euryhaline and commercially important species. MiRNAs participate in the regulation of many physiological activities.

**Results:**

The miRNA transcriptome of the gills of *S. paramamosain* was used to investigate the expression profiles of miRNAs in response to a sudden drop in salinity. In total, seven known miRNAs and 43 novel miRNAs were identified, with 18 differentially expressed small RNAs. Fourteen thousand nine hundred fifty-one differentially expressed miRNAs target genes were screened by prediction. GO analysis of differentially expressed miRNAs target genes indicated that 578 genes associated with cellular processes, 523 associated with metabolic processes, and 422 associated with single-organism processes were the most strongly affected by a sudden drop in salinity from 23‰ to 3‰. KEGG pathway analysis showed 14 pathways were related to amino acid metabolism, which plays an important role in osmoregulation. Besides, several pathways were associated with starch and sucrose metabolism (ko00500), glycosaminoglycan degradation (ko00531), and galactose metabolism (ko00052).

**Conclusions:**

*S. paramamosain* regulated osmotic pressure and energy balance by regulating target genes to adapt to a sudden changes in salinity. These results provided a basis for further investigations of miRNA-modulating networks underlying the osmoregulation of *S. paramamosain*.

**Electronic supplementary material:**

The online version of this article (10.1186/s12864-018-4981-6) contains supplementary material, which is available to authorized users.

## Background

The commercially important mud crab *Scylla paramamosain* (Crustacea: Decapoda: Portunidae: *Syclla De Hann*) is widely distributed along the coasts of southern China and other Indo-Pacific countries [23, 43, 47]. Due to its rapid growth, large size, popularity amongst consumers, and high market value, *S. paramamosain* is becoming an important farmed fish along the southeast coast of China [52, 28]. Mud crab production was 240,293 t in 2016 [10]. Salinity is a key abiotic parameter that influences the distribution, abundance, physiology, and well-being of crustaceans [4, 24, 33]. Although a euryhaline species, a sudden decrease in salinity, especially exceeding 10%, is often lethal to *S. paramamosain*. Previous studies have revealed that crustaceans regulate hemolymph osmotic pressure mainly by adapting to changes in salinity through ion channels [33] and free amino acids [35].

Small noncoding ribonucleic acid (snRNA), which include microRNA (miRNA), small interfering RNA (siRNA), and Piwi-associated RNA (piRNA), has been isolated from a wide variety of plant and mammalian cells. snRNA is involved in various biological and cellular processes by targeting specific miRNAs for degradation or translational repression [3, 8, 21, 48]. So far, most studies have focused on miRNAs, which are 19–23 nucleotides (nt) in length and formed as a single strand of RNA and highly phylogenetically conserved across almost all species [7, 32, 37, 49]. The molecular mechanisms of miRNAs underlying the silencing of target genes have been described [5, 7].

MiRNAs participate in the regulation of many physiological activities [7], including development [12, 15], immunity [18, 31], cell proliferation [6, 54], cell apoptosis [11, 41], and lipid metabolism [40], among others. Many studies have investigated the roles of miRNAs in aquatic animals, including *Oreochromis niloticus* [39], *Oncorhynchus mykiss* [29], *Cyprinus carpio* [27], *Crassostrea gigas* [56], and *Chlamys farreri* [9]. A large number of miRNAs of several aquatic crustaceans, including *Daphnia magna* [42], *Marsupenaeus japonicus* [34], *Litopenaeus vannamei* [50], *Eriocheir sinensis* [14], *S. paramamosain* [22], and *Portunus trituberculatus* [26], have been sequenced and mined.

In our previous study, transcriptomic analysis was conducted on the gills (an important organ in osmoregulation of marine crustaceans) of *S. paramamosain* to reveal the adaptive mechanisms in response to sudden salinity drop under normal conditions (23‰ salinity) and stress conditions caused by a sudden drop in salinity (3‰) [44]. The functional genomic studies of DEGs obtained from Wang et al., [44] allow for a better understanding of various physiological responses in marine crustaceans induced by a sudden drop in salinity. As we all know, microRNAs play a very important role in regulating the expression of functional genes. Therefore, on the basis of previous research, the miRNA transcriptome of the gills of *S. paramamosain* was examined under normal conditions (23‰ salinity) and stress conditions caused by a sudden drop in salinity (3‰) in this study. These results provide a basis for further investigations of miRNA-modulation of networks underlying osmoregulation of *S. paramamosain*.

## Methods

### Animals and salinity challenge experiment

A total of 300 randomly selected crabs with an average body weight of ~ 30 g were selected and maintained in a natural water environment with a salinity of 23‰ and a temperature of ~ 20 °C. The crabs were randomly assigned to one of six groups of 50 animals each and housed in six cement pools under identical physical and chemical conditions. The salinity of the seawater for three of the groups was adjusted to 3‰ from 23‰, and then decreased to 20‰. These three groups were defined as the LS (low salinity) group. The other three groups were defined as the CK groups, where the salinity of seawater was maintained at 23‰. All other conditions were the same as the LS group.

According to [44], there were four deaths in the CK group within 7 days and 24 deaths in the LS group. The LS death time was concentrated within 24, 48, and 72 h. In addition, the LS group showed hyperactivity within 48 h, and as time went by, the motility was diminished and normalized. The LS group did not have food over 72 h, and gradually started to eat over time. Conditions returned to normal after 120 h. In our study, the mud crabs had adjusted to a salinity of 3‰ in 120 h after a sudden drop in salinity. In order to study the molecular mechanism underlying this adaptation, we performed transcriptional profiling at 120 h [44]. The crabs in CK and LS group at 120 h were killed under the condition of alcohol anesthesia, and gills were quickly removed and used for subsequent sequencing and studies.

### The construction and deep sequencing of small RNA libraries

Total RNA was extracted from *S. paramamosain* gills tissues with TRIzol reagent (Invitrogen Corporation, Carlsbad, CA, USA), as recommended by the manufacturer.

RNA fragments, 18–30 bases in length, were isolated from total RNA extracted from gills of three individual mud crabs as one sample after being separated through 15% denaturing gels to net three samples from each group. Then, sRNAs were excised from the gel and sequentially ligated to 3′- and 5′-adapters. After each ligation step, sRNAs were separated in 15% denaturing gels. The final purified ligation products were reversely transcribed into complementary DNA (cDNA) using reverse transcriptase (Finnzymes Oy, Espoo, Finland). The first strand cDNA was PCR amplified using Phusion* DNA polymerase (Finnzymes Oy). The purified DNA fragments were used for clustering and sequencing using the Illumina Hiseq 4000 sequencing system (Illumina, Inc., San Diego, CA, USA), which was performed by BGI Diagnosis Technology Co., Ltd. (Shenzhen, China).

### miRNA identification

Raw data (raw tags) in the FASTQ format were first processed using self-written Perl and Python scripts (BGI, Shenzhen, China). In this step, clean data (clean tags) were processed by removing low-quality tags containing ploy-N, with 5′ primer contaminants, without 3′ primers or the insert tag, and with ploy A or T or G or C from the raw data. At the same time, the Q20, Q30, and GC contents of the raw data were calculated. Then, the length of sRNA tags within a certain range from clean tags was determined for downstream analysis. All clean tags were searched against the Rfam database (http://rfam.xfam.org//) for annotation. The tags annotated as tRNA, rRNA, small nucleolar RNA (snoRNA), and snRNA were discarded. Considering that there was no available information of the *S. paramamosain* genome, the remaining small RNA tags were compared to known miRNAs from all metazoan species in the miRBase 21.0 database (http://www.mirbase.org/) to identify conserved miRNAs. Only perfect matches were considered as conserved miRNAs. Tags that were not aligned to the miRBase database were used to predict novel miRNAs. The miREvo evolutionary analysis platform for next-generation sequencing and mirdeep2 software package were used to predict novel miRNAs by exploring the secondary structure, the Dicer cleavage site, and the minimum free energy of the former unannotated small RNA tags that could be mapped to reference sequences. Q_value < 0.001 and |log2 (foldchange) | > 1 were set as thresholds to identify significantly differential expression of miRNAs.

### Target gene prediction and analysis

As there was no published information on the *S. paramamosain* genome, the assembled Unigenes from the *S. paramamosain* (https://www.ncbi.nlm.nih.gov/sra/SRP129841, SRA accession: SRP129841; Temporary Submission ID: SUB3501735) [44] were considered as candidate genes for target gene prediction. The RNAhybrid [20], miRanda [17], and TargetScan [2] web servers were used to predict the target genes of identified miRNAs. The predicted target genes were aligned using the Basic Local Alignment Search Tool (https://blast.ncbi.nlm.nih.gov/). Afterward, gene ontology (GO) analysis of the target genes was performed using the Gene Ontology Enrichment Analysis Software Toolkit (http://omicslab.genetics.ac.cn/GOEAST/; [55]).

### Quantitative real-time PCR assay

MiRNAs expression levels were assayed by quantitative realtime PCR (qPCR) using a SYBR primescript™ miRNA RT-PCR kit (TaKaRa). Total RNA was isolated from the gills of mud crabs in the LS groups at 120 h after a sudden drop in salinity from 23‰ to 3‰ using RNAiso Plus total RNA extraction reagent (TaKaRa), while total RNA was isolated at 120 h from crabs in the CK groups maintained at a salinity of 23‰. The cDNA was synthesized using the Perfect Real Time version of the PrimerScript™ RT reagent kit with gDNA Eraser (Perfect Real Time) (TaKaRa) according to the manufacturer’s instructions. The 18S rRNA gene was selected as an internal control. Primers used in this study are listed in Table 1. The qPCR was conducted in 15 μl reaction volumes containing 300 nM of each primer and cDNA derived from 0.1 μg of total RNA. Cycling parameters were 95 °C for 2 min, and followed by 50 cycles of 95 °C for 10 s, 60 °C for 10 s and 72 for 40 s. All reactions were run in triplicate. The 2 ^-ΔΔCT^ method was employed in the analysis of relative quantification. Significant differences were examined by paired t-test in which *p* value< 0.01 and *p* value< 0.01 was considered to be statistically significant.

**Table 1:** Primers for RT-qPCR amplification of miRNAs

ID	Primer	Sequence (5′-3′)
miR-7	RT	GTCGTATCCAGTGCGTGTCGTGGAGTCGGCAATTGCACTGGATACGACCAACAAA
	Forward	GTGGAAGACTAGTGATTTTG
	Reverse	TGCGTGTCGTGGAGTC
novel_mir16	RT	GTCGTATCCAGTGCGTGTCGTGGAGTCGGCAATTGCACTGGATACGACTCTGAAC
	Forward	GCACCGAAGCTTAGGGTT
	Reverse	TGCGTGTCGTGGAGTC
novel_mir18	RT	GTCGTATCCAGTGCGTGTCGTGGAGTCGGCAATTGCACTGGATACGACAGAATAC
	Forward	GCCTATAATGGCTATTGGTA
	Reverse	TGCGTGTCGTGGAGTC
novel_mir19	RT	GTCGTATCCAGTGCGTGTCGTGGAGTCGGCAATTGCACTGGATACGACGCATCT
	Forward	ATCCTTGGACCACAGCAG
	Reverse	TGCGTGTCGTGGAGTC
novel_mir22	RT	GTCGTATCCAGTGCGTGTCGTGGAGTCGGCAATTGCACTGGATACGACACACCT
	Forward	TGAGGGTGACTGGCAGG
	Reverse	TGCGTGTCGTGGAGTC
novel_mir24	RT	GTCGTATCCAGTGCGTGTCGTGGAGTCGGCAATTGCACTGGATACGACTTCGGC
	Forward	CACCACTCTTGTCTCTGC
	Reverse	TGCGTGTCGTGGAGTC
novel_mir26	RT	GTCGTATCCAGTGCGTGTCGTGGAGTCGGCAATTGCACTGGATACGACTCGAGT
	Forward	ATGATGGCAGCGGTGACT
	Reverse	TGCGTGTCGTGGAGTC
novel_mir31	RT	GTCGTATCCAGTGCGTGTCGTGGAGTCGGCAATTGCACTGGATACGACACTGAC
	Forward	CTAATTTGAGCCATCTGTCA
	Reverse	TGCGTGTCGTGGAGTC
novel_mir34	RT	GTCGTATCCAGTGCGTGTCGTGGAGTCGGCAATTGCACTGGATACGACCAAGATG
	Forward	CACAGCCGTGTAGTCATC
	Reverse	TGCGTGTCGTGGAGTC
novel_mir35	RT	GTCGTATCCAGTGCGTGTCGTGGAGTCGGCAATTGCACTGGATACGACTGGCCA
	Forward	GTATTGGGCGTGTGTTGG
	Reverse	TGCGTGTCGTGGAGTC
.novel_mir37	RT	GTCGTATCCAGTGCGTGTCGTGGAGTCGGCAATTGCACTGGATACGACTGAGGA
	Forward	TCGCAGATCCAGAATGTTC
	Reverse	TGCGTGTCGTGGAGTC
novel_mir40	RT	GTCGTATCCAGTGCGTGTCGTGGAGTCGGCAATTGCACTGGATACGACACTGAAG
	Forward	TGGAATGCATGGCTACACT
	Reverse	TGCGTGTCGTGGAGTC
novel_mir45	RT	GTCGTATCCAGTGCGTGTCGTGGAGTCGGCAATTGCACTGGATACGACTGTCCG
	Forward	GGCGTGGCAGGGGTTTC
	Reverse	TGCGTGTCGTGGAGTC
novel_mir47	RT	GTCGTATCCAGTGCGTGTCGTGGAGTCGGCAATTGCACTGGATACGACTCGAGT
	Forward	ATGATGGCAGCGGTGACT
	Reverse	TGCGTGTCGTGGAGTC
novel_mir48	RT	GTCGTATCCAGTGCGTGTCGTGGAGTCGGCAATTGCACTGGATACGACACAGGC
	Forward	TACCCTGATATTCCTTGCC
	Reverse	TGCGTGTCGTGGAGTC
novel_mir5	RT	GTCGTATCCAGTGCGTGTCGTGGAGTCGGCAATTGCACTGGATACGACTTTGGGA
	Forward	TCAATGCCCTTGGAAATCC
	Reverse	TGCGTGTCGTGGAGTC
novel_mir53	RT	GTCGTATCCAGTGCGTGTCGTGGAGTCGGCAATTGCACTGGATACGACCTTAGG
	Forward	GGGTTAGTCGGGTCCTA
	Reverse	TGCGTGTCGTGGAGTC
novel_mir7	RT	GTCGTATCCAGTGCGTGTCGTGGAGTCGGCAATTGCACTGGATACGACTCTCACA
	Forward	GATGACTACACGGCTGTG
	Reverse	TGCGTGTCGTGGAGTC
18S rRNA	Forward	GGAATTCCCAGTAAGCGCAA
	Reverse	CCAGTCCGAAGGCCTCACTA

18S rRNA: a reference gene of the *S. paramamosain*

## Results

### Bgiseq-500 sequencing of small RNAs

**Fig. 1:**
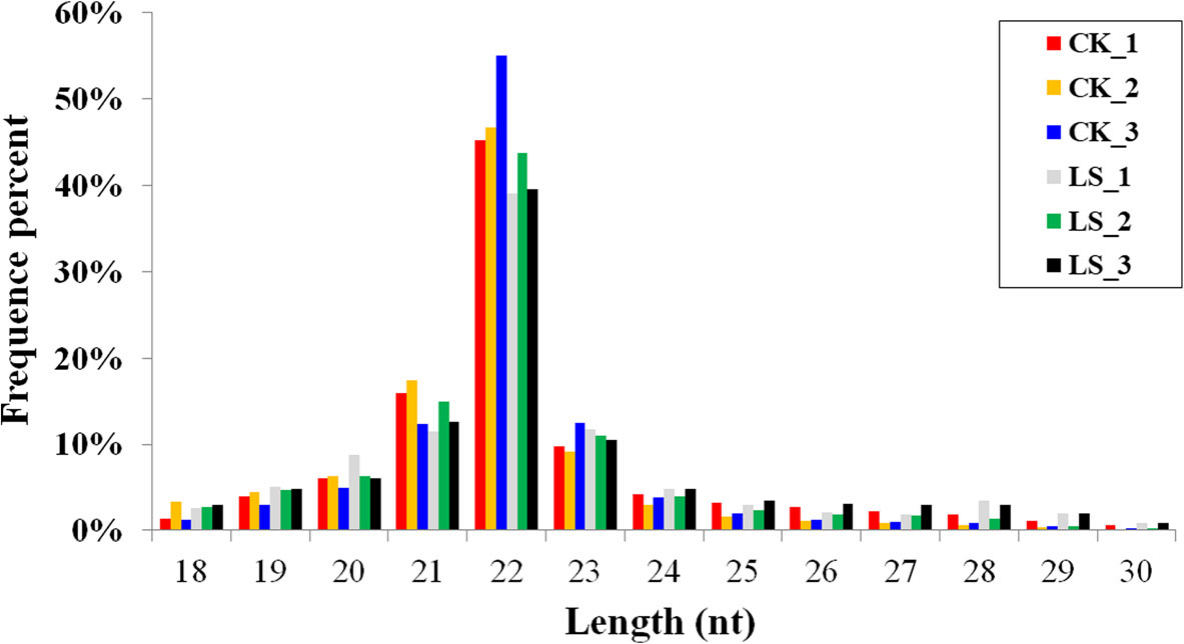
The distribution of small RNAs relative to length in the CK and LS groups

To identify miRNAs of the mud crab in response to a sudden drop in salinity, small RNA libraries derived from the CK and LS groups were constructed and sequenced using the BGIseq-500 Next Generation Sequencing Platform. Raw tags were obtained from the three CK groups and three LS groups, respectively (Table 2). After removal of low quality sequences, contaminated with 5′ linkers, no 3′ linker sequence, no inserts, poly A included, shorter than 18 nt in length, clean tags (Table 2) of 18–30 nt in length were obtained. The clean tags of the CK and LS libraries were similar in size distribution and frequency, and most of the sequences were 21–23 nt in length (Fig. 1), of which those 22 nt in length were the most abundant (Fig. 1). After data filtering, the clean tags were compared with known small RNA databases, including miRBase [19], Rfam [30], siRNA, piRNA, snoRNA, etc. Other non-coding RNAs (rRNA, snoRNA, tRNA, and snRNA) and repeat sequences (Table 3) were annotated. The remaining tags of the CK and LS libraries were used for miRNA analysis, respectively.

**Table 2:** Statistics of small RNAs BGISEQ-500 sequencing

Samples	Total raw tags	Total clean tags	Mapped tag	Q20 (%)	GC (%)
CK_−_1	34,356,213	32,141,147	25,947,876	98.90	49.80
CK_−_2	33,684,193	30,152,225	29,836,952	99.30	48.20
CK_−_3	33,284,649	30,658,107	19,951,162	99.40	49.40
LS_−_ 1	34,564,325	31,352,016	20,143,843	98.90	51.90
LS_−_2	33,142,424	29,484,837	20,538,317	99.20	50.30
LS_−_3	32,660,103	29,836,952	22,220,048	99.30	53.90

*Sample* Sample name, *Total raw tags* the total number raw data of small RNAs, *Total clean tags* the total number clean tags of small RNAs, *Mapped tag* the number of tags matched to the genome, *Q20 (%)* the number of base calls with quality value of 20 or higher (Q20+) (%), *GC (%)* the percentage of G and C bases in the small RNAs

**Table 3:** Annotation of small RNAs

Sample name	known_miRNA	novel_miRNA	rRNA	snoRNA	tRNA	snRNA	repeat	other	total
CK_−_1	2,100,459	3,133,891	542,451	1692	77,938	11,003	102,088	2,918,607	8,888,129
CK_−_2	2,614,045	2,776,978	139,310	582	20,908	8354	71,225	2,611,725	8,243,127
CK_−_3	4,397,452	5,903,941	252,238	713	33,641	2463	43,649	8,122,265	18,756,362
LS_−_1	3,887,397	4,541,837	253,857	1400	33,878	2553	73,639	5,125,302	13,919,863
LS_−_2	3,290,026	4,541,837	236,226	949	84,760	3637	63,418	4,444,753	12,665,606
LS_−_3	2,034,046	1,910,369	282,526	819	82,212	10,012	84,969	2,172,853	6,577,806

### The discovery of known, novel and differential expression of miRNAs

In the CK and LS libraries, there were a total of seven known miRNAs (miR-100-5p_1, miR-100-5p, miR-100, miR-7a, miR-7a_1, miR-7_2, and miR-7) of variable abundances. MiR-100-5p___1 was the most accumulated miRNA, with a total of 11,848,942 tags from the CK and LS libraries (5,794,803 tags in the CK group and 6,054,139 in the LS group). While the least abundant miRNA in the two libraries was miR-7 (1157 tags in the CK group and 6374 in the LS group) (Table 4).

**Table 4:** miRNA expression in the LS and CK groups

ID	Sequence	Read count		log_2_Ratio (LS/CK)	Up / down	*P*_ value	*Q* _ value	Different expression
		CK	LS					
novel_mir1	AAGAGAGCUAUCCGUCGACAGU	11,244,923	9,582,299	−0.12	down	0	0	No
miR-100-5p_1	AACCCGUAGAUCCGAACUUGU	5,794,803	6,054,139	0.18	up	0	0	No
miR-100-5p	AACCCGUAGAUCCGAACUUGUG	2,939,724	2,764,103	0.03	up	1.68E-127	1.02E-128	No
miR-100	AACCCGUAGAUCCGAACUU	326,542	343,837	0.19	up	0	0	No
novel_mir2	AUAGGUAGCUCUGAGUCCAGAG	217,367	216,078	0.11	up	1.4E-133	9.19E-135	No
novel_mir3	UACUGGCCUGCUAAGUCCCAAG	105,094	113,448	0.23	up	2.12E-295	2.58E-296	No
novel_mir4	UCCCUGAGACCCUUUCUUGUGA	45,082	50,918	0.29	up	3.49E-215	2.97E-216	No
novel_mir8	AAAUAUCAGCUGGUAAAUUUGG	23,653	28,276	0.37	up	1.1E-190	7.78E-192	No
novel_mir5	CAAUGCCCUUGGAAAUCCCAAA	55,060	25,181	−1.01	down	0	0	Yes
miR-7a	UGGAAGACUAGUGAUUUUGUUGUU	22,817	23,572	0.16	up	5.26E-34	2.49E-35	No
novel_mir7	AGAUGACUACACGGCUGUGAGA	67,340	20,469	−1.60	down	0	0	Yes
novel_mir6	UGACUAGAGAUUCACACUCAUCC	17,884	18,990	0.20	up	2.25E-41	1.2E-42	No
novel_mir29	UGAACACAGCUGGUGGUAUCU	14,482	18,625	0.48	up	5.76E-199	4.45E-200	No
miR-7a_1	UGGAAGACUAGUGAUUUUGUUGU	12,463	11,125	−0.05	down	0.010473726	0.00024088	No
novel_mir9	CUUACGACCGCCUAGCACGGUA	8913	8789	0.10	up	0.0000104	2.77E-07	No
miR-7_2	UGGAAGACUAGUGAUUUUGUU	14,450	8319	−0.68	down	7.7E-268	8.19E-269	No
novel_mir10	GUACCGAAGCUGCGGAUGCGU	4672	8016	0.89	up	4.01E-258	3.79E-259	No
miR-7	UGGAAGACUAGUGAUUUUGUUG	1157	6374	2.58	up	0	0	Yes
novel_mir11	UGAGAGUGAGAGAUAGAGAGGA	5282	5626	0.21	up	7.06E-14	2.86E-15	No
novel_mir46	UGAUACUCGGGUGCCUGUUC	1227	1642	0.54	up	4.03E-23	1.81E-24	No
novel_mir56	GGUGUAGCAUAAGUGGGA	1384	975	−0.39	down	7.45E-11	2.75E-12	No
novel_mir39	UCUCCUCUUCCACUUUCUCGUC	245	415	0.88	up	1.71E-14	7.28E-16	No
novel_mir42	GUACAUGAGUUUGGGGAGGAUG	412	410	0.11	up	0.279773023	0.00529043	No
novel_mir12	AGAGGAAGCACAGGAUGAAGCA	299	321	0.23	up	0.059704709	0.00123915	No
novel_mir16	GCACCGAAGCUUAGGGUUCAGA	31	247	3.11	up	3.65E-46	2.07E-47	Yes
novel_mir13	UGAGGGGAAUGUGUUGGCCAGU	211	205	0.07	up	0.600140943	0.01063922	No
novel_mir34	UCACAGCCGUGUAGUCAUCUUG	536	172	−1.52	down	9.21E-39	4.61E-40	Yes
novel_mir38	UAACGUUUCGUACAGAGUACUU	86	156	0.97	up	0.000000268	8.45E-09	No
novel_mir25	CCUAUCACCACUACCACUACUG	118	106	−0.04	down	0.839846761	0.01429315	No
novel_mir17	UGGUGGCUGGUCGAGCGAGGACU	62	72	0.33	up	0.184002039	0.00369166	No
novel_mir21	UGGAGGAUGGAAGGCCGUGUGU	74	54	−0.34	down	0.186548791	0.00369166	No
novel_mir27	GUCGAGGAGAGGUCAGUGCCA	28	50	0.95	up	0.004289186	0.00010138	No
novel_mir47	AUGAUGGCAGCGGUGACUCGA	0	43	6.54	up	4.94E-12	1.91E-13	Yes
novel_mir36	ACGGGUGGAUGGGUGGGUG	37	43	0.33	up	0.303079488	0.00560656	No
novel_mir50	CGGGAGAGUUAAUUAGCAGUGUU	65	41	−0.55	down	0.053070425	0.00112899	No
novel_mir22	UGAGGGUGACUGGCAGGUGU	0	32	6.12	up	0.000000002	6.82E-11	Yes
novel_mir26	AUGAUGGCAGCGGUGACUCGA	14	28	1.12	up	0.014920708	0.00033412	Yes
novel_mir40	UGGAAUGCAUGGCUACACUUCAGU	0	27	5.87	up	3.44E-08	1.12E-09	Yes
novel_mir53	GGGUUAGUCGGGUCCUAAG	14	27	1.06	up	0.021514563	0.00046942	Yes
novel_mir49	UUGGCUGAUCCAGUAAGUUGU	40	27	−0.45	down	0.2046118	0.00395709	No
novel_mir35	UGUAUUGGGCGUGUGUUGGCCA	0	23	5.64	up	0.000000354	1.08E-08	Yes
novel_mir18	UAUAAUGGCUAUUGGUAUUCU	0	14	4.92	up	0.0000862	0.00000222	Yes
novel_mir20	UGACGGCGCCGCCACUACUGCU	17	14	−0.16	down	0.75159718	0.0130523	No
novel_mir19	AUCCUUGGACCACAGCAGAUGC	0	12	4.70	up	0.000309356	0.00000774	Yes
novel_mir37	CGCAGAUCCAGAAUGUUCCUCA	37	12	−1.51	down	0.000684211	0.0000166	Yes
novel_mir41	CGUGGGCAGGUGUGGGUGGCU	16	12	−0.30	down	0.584887298	0.01058942	No
novel_mir45	GGCGUGGCAGGGGUUUCUCGGACA	37	0	−6.09	down	5.77E-10	2.04E-11	Yes
novel_mir24	UCACCACUCUUGUCUCUGCCGAA	24	0	−5.47	down	0.0000006	1.76E-08	Yes
novel_mir48	UUACCCUGAUAUUCCUUGCCUGU	23	0	−5.41	down	0.00000105	2.96E-08	Yes
novel_mir31	CUAAUUUGAGCCAUCUGUCAGU	21	0	−5.28	down	0.0000032	8.79E-08	Yes

RNA sequencing was conducted as a random sampling process, in which each read was sampled independently and uniformly from every possible nucleotide in the sample [16]. Under this assumption, the number of reads of a single gene (or transcript isoform) follows a binomial distribution (and can be approximated by a Poisson distribution). Screening differentially expressed small RNAs [45] proposes a novel method based on the MA-plot, which is a statistical analysis tool having been widely used to detect and visualize the intensity-dependent ratio of microarray data [51]. ID: miRNA id; CK: reads count of sample CK; LS: reads count of sample LS; log_2_Ratio (LS/CK): Log2 of the difference multiple; Up / down: Up (down) regulated

A total of 43 novel miRNAs were identified in the CK and LS libraries, and displayed unequal expression levels (Table 4). The top five most abundant miRNAs were novel_mir1, miR-100-5p_1, miR-100-5p, miR-100, and novel_mir2, with 20,827,222, 11,848,942, 5,703,827, 670,379 and 433,445 tags detected between the two libraries. Besides, novel_mir18, novel_mir19, novel_mir22, novel_mir35, novel_mir40 and novel_mir47 were found only in the CK groups, while novel_mir45, novel_mir24, novel_mir48, and novel_mir31 were only found in the LS groups, with read counts all below 40 tags. The miRNAs of *S. paramamosain* were matched against the miRNAs of *Portunus trituberculatus*. The salinity regulation -related miRNAs in gills included mir_7 (known), novel_mir1, novel_mir4, and novel_mir8.

A comparison of miRNA expressed in response to a sudden drop in salinity (Q___value< 0.001 and |log_2_ (fold change)| > 1), indicated that a total of 18 miRNAs were differentially expressed small RNAs (DESs), with 10 miRNAs up-regulated and eight down-regulated (Table 4 and Additional file 1: Table S1). In the CK and LS libraries, the most up-regulated miRNA was novel_mir47 (log_2_.Fold_change = 6.542014656) and the most down-regulated miRNA was novel_mir45 (log_2_ Fold_change = − 6.093703464). Among the 18 miRNAs, the most abundant miRNAs were novel_mir7, novel_mir5, and miR-7 with 87,809, 80,241, and 7531 tags detected between the CK and LS libraries, respectively (Table 4).

### Validation and target gene prediction of differentially expressed miRNAs

**Fig. 2:**
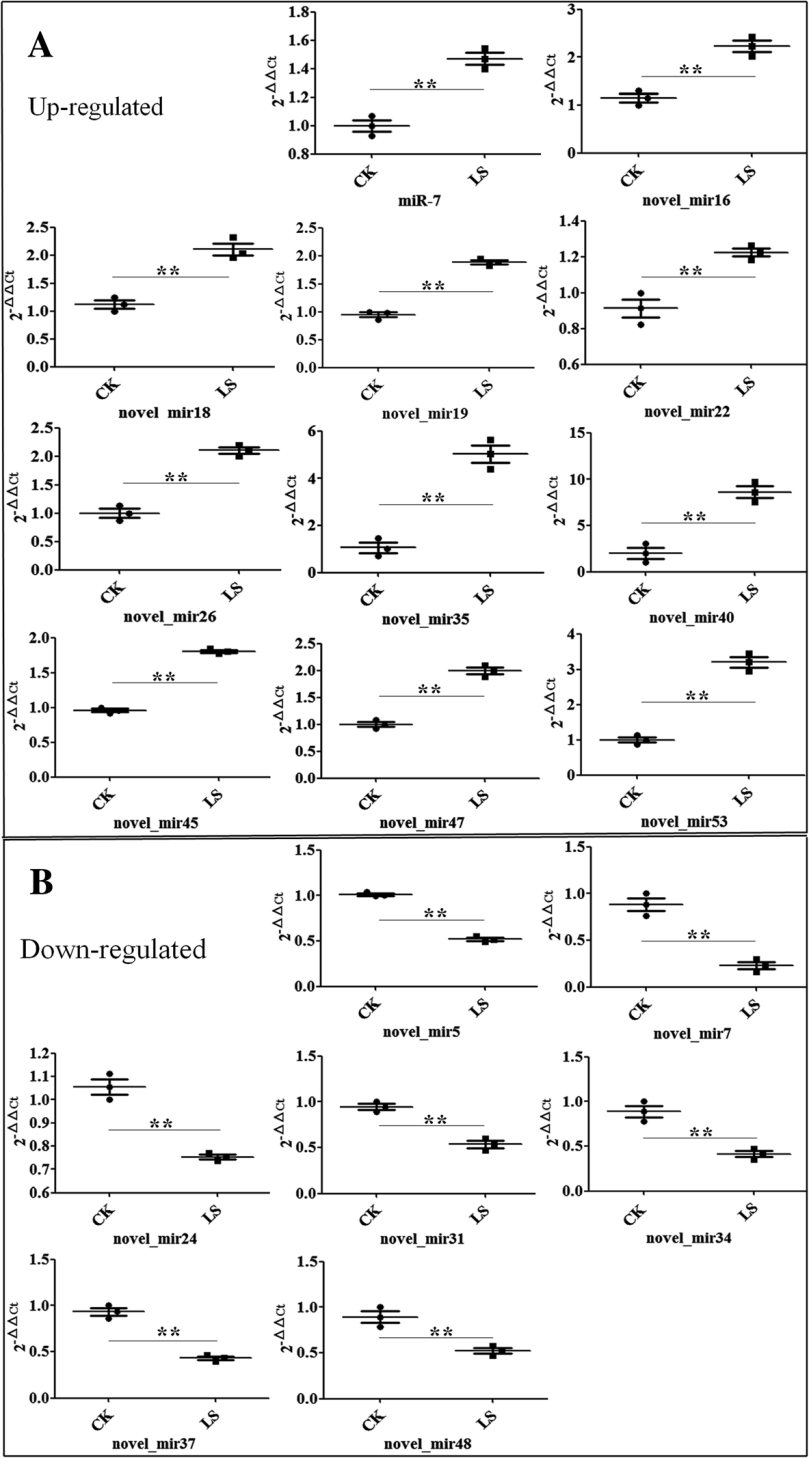
Gene expression levels of all of 18 differentially expressed miRNAs for the comparison of CK and LS groups according qPCR analysis. **a** Up-regulated differentially expressed miRNAs; **b** Down-regulated differentially expressed miRNAs. The significant difference of miRNA expression between CK and LS was indicated with asterisks (**: *P* < 0.01)

QPCR was adopted to validate the 18 differentially expressed miRNAs acquired from sequencing. The results showed that miR-7, novel_miR16, novel_miR18, novel_miR19, novel_miR22, novel_miR26, novel_miR35, novel_miR40, novel_miR45, novel_miR47 and novel_miR53 were differentially expressed up-regulated miRNAs (Fig. 2a & Additional file 1: Table S2), with ratio (LS / CK) ranging from 1.34 to 4.73; meanwhile, novel_miR5, novel_miR7, novel_miR24, novel_miR31, novel_miR34, novel_miR37 and novel_miR48 were differentially expressed down-regulated miRNAs, with ratio (CK / LS) ranging from 1.40 to 3.86 (Fig. 2b, Additional file 1: Table S2). The differences of the 18 differentially expressed miRNAs all reached the extremely significant level (P<0.01) (Fig. 2). The genes changes most according to qPCR results were novel_mir35 and novel_mir24 (Additional file 1: Table S2), among the up-regulated and down-regulated miRNAs, respectively. Though the trend (up or down) was nearly same as sequencing results (Additional file 1: Tables S1 & S2), the specific figure were different from each other. Perhaps because the sensitivity of the two different detection methods brought about the differences between sequencing and qPCR results. Moreover, novel_mir45 was down-regulated in sequencing results (Additional file 1: Table S1), but up-regulated in qPCR results (Fig. 2a & Additional file 1: Table S2). The reason was unknown.

**Fig. 3:**
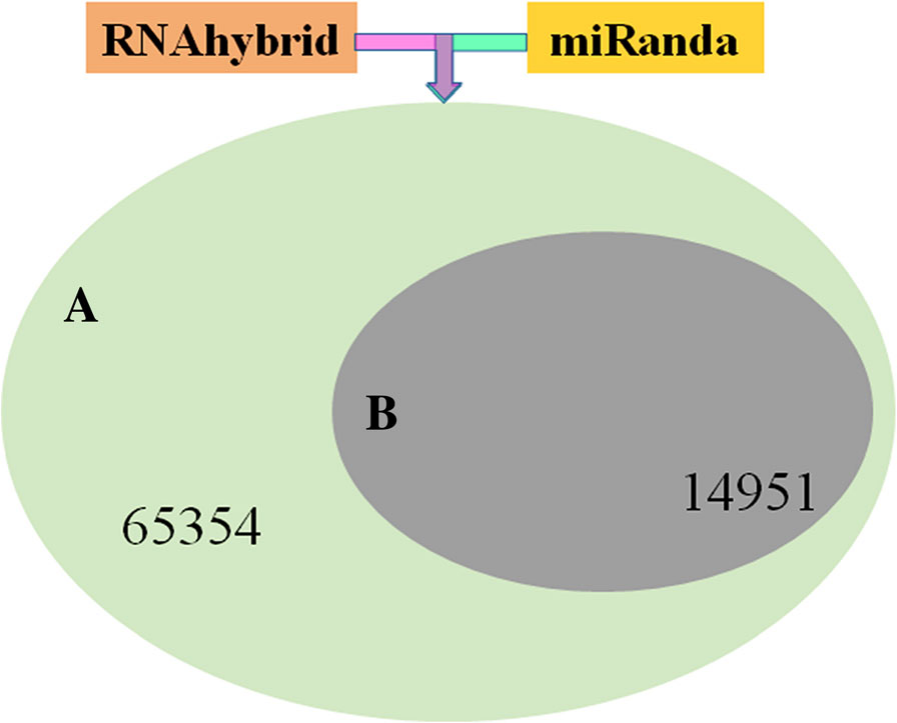
Target prediction by RNAhybrid and miRanda. **a** Statistics of total miRNAs target prediction. **b** Statistics of differentially expressed miRNAs target prediction

Using RNAhybrid [20], the microRNA Target Prediction Tool (miRanda, [17]) was used to predict the target genes of miRNAs, combined with corresponding filtering conditions, such as free energy, score values, etc., to obtain an aggregation of the target genes of the identified miRNAs. A total of 65,354 unigenes of the *S. paramamosain* transcriptome were identified as miRNA targets (Fig. 3a), and 18 differentially expressed miRNAs had 14,951 target genes (Fig. 3b).

### GO classification, and KEGG pathway analysis of differentially expressed miRNAs target genes

**Fig. 4:**
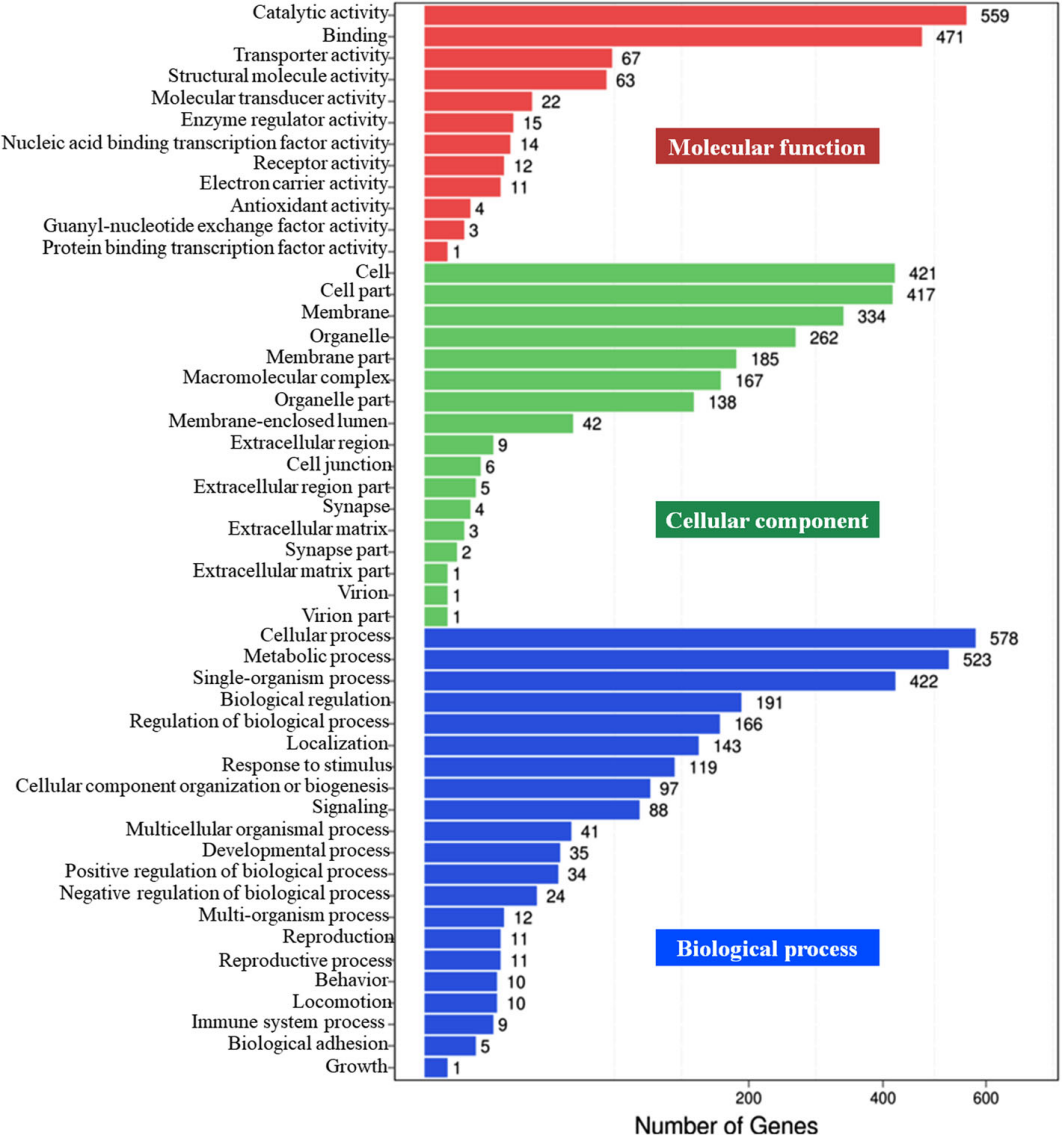
GO functional classification of differentially expressed miRNAs target genes from the gill of *S. paramamosain* in response to a sudden drop in salinity from 23‰ to 3%*.* The number of DEGs (presented as a square root value) is shown on the X axis, while GO terms are shown on the Y axis. All GO terms are grouped in to three ontologies: biological processes are shown in blue, cellular components in green, and molecular functions in red

GO functional classification of 18 differentially expressed miRNAs target genes was performed using WEGO software [53]. According to the second-tier GO terms, there were three main GO categories: biological process (21), cellular component (17), and molecular function (12) (Fig. 4). In total, 21 processes were identified in the biological process category, with 578 DESs of target genes involved in cellular processes, 523 involved in metabolic processes, and 422 involved in single-organism processes (Fig. 4). These three biological processes were the most strongly affected in the gill of *S. paramamosain* by a sudden drop in salinity from 23‰ to 3‰ (Fig. 4). In the cellular component category, cell (421), cell parts (417), membrane (334), and membrane part (262) were the most common (Fig. 4). In the molecular function category, catalytic activity (559) and binding (471) were the most common (Fig. 4).

**Fig. 5:**
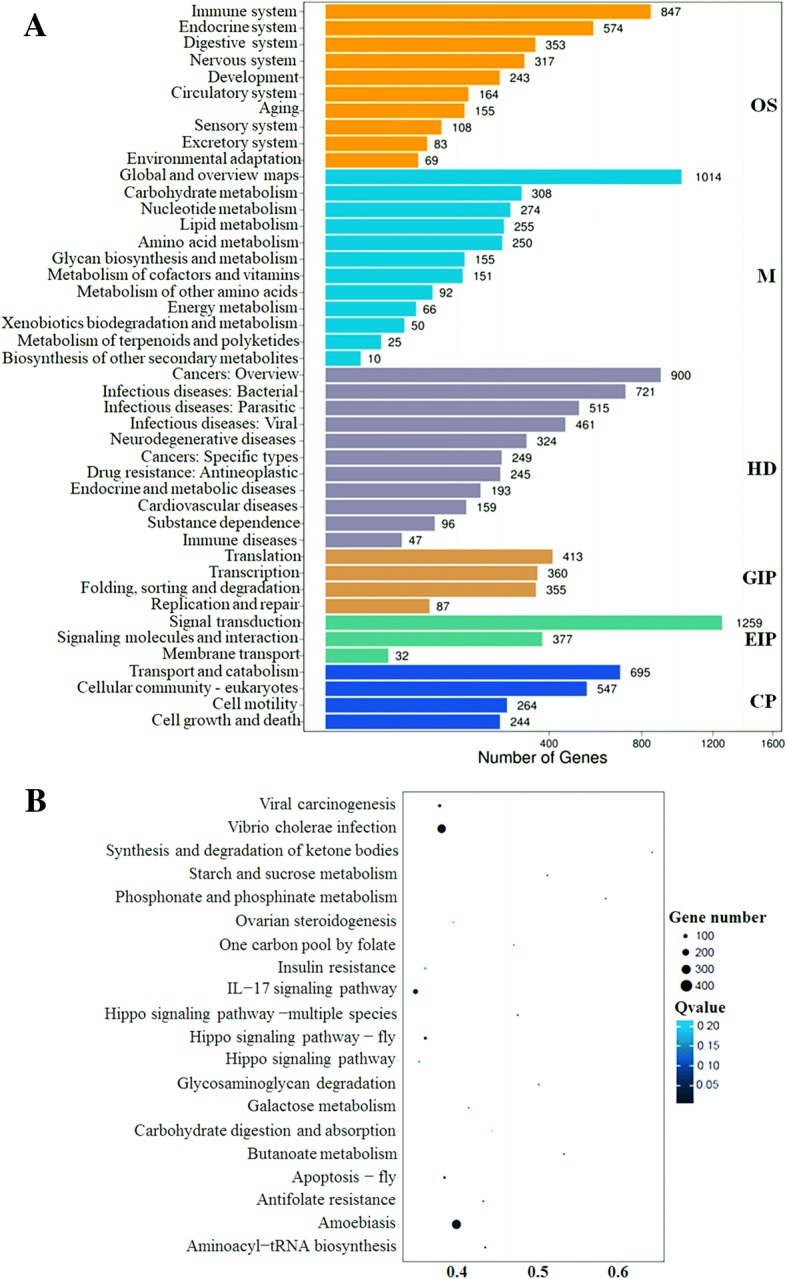
KEGG pathway classification (**a**) and functional enrichment (**b**) of differentially expressed miRNAs target genes. **a** The numbers of DEGs are shown on the X axis, while the KEGG pathway terms are shown on the Y axis. All second pathway terms are grouped in the following top pathway terms, as indicated by different colors: OS, organismal systems; M, metabolism; HD, human diseases; GIP, genetic information processing; EIP, environmental information processing; and CP, cellular processing. **b** RichFactor is the ratio of DESs of target genes annotated in this pathway term to all gene numbers annotated in this pathway term. Greater richFator means greater intensiveness. The Q-value is the corrected *p*-value ranging from 0~ 1. A lower Q-value indicates greater intensiveness. The top 20 enriched pathway terms are displayed (Q-value < 0.05)

KEGG pathway classification of differentially expressed miRNAs target genes was performed. Of all DESs of the target genes, 5019 were mapped in 319 KEGG pathways (Additional file 1: Table S2), which were classified into six categories (level 1) according to their biological functions, which included organismal systems, human diseases, environmental information processing, genetic information processing, metabolism, and cellular processes (Fig. 5a). Of the 319 pathways, 91 (28.53%), 79 (24.76%), 76 (23.82%), 34 (10.66%), 22 (6.90%), and 17 (5.33%) were related to metabolism, organismal systems, human diseases, environmental information processing, genetic information processing, and cellular processes, respectively (Fig. 5a and Additional file 1: Table S3). /Pathway enrichment analysis of the DESs of the target genes based on the top 20 of KEGG enrichment results (Fig. 5b and Additional file 1: Table S3) showed the largest number of pathways were related to metabolism, such as starch and sucrose metabolism (ko00500), glycosaminoglycan degradation (ko00531), and galactose metabolism (ko00052), which are closely related to energy metabolism. These results suggest that cell metabolism in the gill of *S. paramamosain* was altered tremendously in response to a sudden drop in salinity from 23‰ to 3%. Especially, glycometabolism associated closely with energy metabolism was enhanced. So changes in cell metabolism in the gills of mud crab were very important for adaptation to a sudden drop in salinity to maintain physiological activities.

## Discussion

Most crustaceans live in aquatic environments and are very sensitive to chemical changes in water. Changes in water salinity directly affect the growth, survival rate, and reproduction of crustaceans, which are mainly related to changes in osmotic pressure and ion concentrations in body fluids. Artificial breeding environment restrictions (i.e., mass water exchange and heavy rainfall) and desalinization will lead to environmental salinity-induced stress. A rapid change in salinity due to heavy rainfall and mass water exchange will cause acute stress, while long-term decrease in salinity of an estuary can cause chronic stress, although crustaceans can adapt to such low-salt environments. Most euryhaline crustaceans can maintain internal homeostasis. The internal balance mechanisms (compensatory reactions) of euryhaline crustaceans can maintain homeostasis in response to a sudden change in salinity. This process can be simplified as a change from “stress” to “adaptation” without “failure.” However, high-intensity salinity stress may result in serious adverse reactions of crustaceans, including reduced production, morbidity, or even death. *S. paramamosain* is an economical euryhaline large-sized marine crustacean. The salinity of water for culturing of *S. paramamosain* is generally < 25‰ and the lowest salinity is close to freshwater (such as around Shanghai, China). Mud crabs are mainly distributed throughout estuary areas with large changes in salinity. During the rainy season, the salinity of farm ponds greatly vary, which can adversely affect the culture of *S. paramamosain*, resulting in sever economic losses. Because *S. paramamosain* is a euryhaline species, its sensitivity to sudden changes of salinity is often overlooked. This study preliminarily explored the adaptive mechanism of *S. paramamosain* to a sudden drop in salinity via changes in miRNA profiles of the gills. It is worth noting that the gills of *S. paramamosain* in the LS group had adapted to the change in salinity to 3‰ for 120 h.

Previous studies have shown that miRNAs are widely involved in various biological processes in animals, including cell differentiation, proliferation, development, apoptosis, metabolism, signal transduction, immunity, and evolution. MicroRNAs can guide silencing complexes to cleave target genes or inhibit the translation of target genes by pairing with 3′UTR region [13, 36]. GO functional classification of the DESs of target genes in response to a sudden drop in salinity from 23‰ to 3% revealed that 91 (28.53%) were related to metabolism. Many pathways were associated with amino acid metabolism, such as degradation of valine, leucine, and isoleucine (ko00280), isoleucine biosynthesis (ko00290), histidine metabolism (ko00340), arginine and proline metabolism (ko00330), tryptophan metabolism (ko00380), arginine biosynthesis (ko00220), alanine, aspartate, and glutamate metabolism (ko00250), glycine, serine, and threonine metabolism (ko00260), lysine degradation (ko00310), cysteine and methionine metabolism (ko00270), tyrosine metabolism (ko00350), lysine biosynthesis (ko00300), phenylalanine metabolism (ko00360), and phenylalanine, tyrosine, and tryptophan biosynthesis (ko00400) (Fig. 5 and Additional file 1: Table S3). Free amino acids play important roles in osmoregulation [1, 25, 46]. The results of the current study implied that amino acid metabolism in the gills of *S. paramamosain* was altered to equilibrate changes in exoteric osmotic pressure. Besides, we also identified pathways closely associated with energy metabolism, such as starch and sucrose metabolism (ko00500), glycosaminoglycan degradation (ko00531), and galactose metabolism (ko00052), among others (Additional file 1: Table S3). Changes in salinity lead to increased rates of oxygen consumption in shrimp and crabs, as well as an increased demand for energy, accelerated metabolism, and physiological dysfunction, which can results in reduced immunity [38]. Because salinity affects respiratory metabolism and energy balance of crustaceans, a sudden change in salinity may lead to a change in the osmotic pressure of body fluids of crustaceans, thereby resulting in energy dysregulation, which requires consumption of additional energy in response to osmotic pressure adjustments.

## Conclusion

Although *S. paramamosain* is an important economical cultured marine animal, few studies have investigated osmotic adjustments in response to a change in salinity. Studies on the capabilities of crustaceans to adapt to different salinities are limited, especially research at the transcriptome and protein levels. However, one such study by the author of this article is currently under review. In this study, high-throughput sequencing technology was used to obtain the miRNA profiles of the gills of a normal control group (23‰) and in response to a sudden drop in salinity (3‰). The results of this study identified seven known miRNAs and 43 novel miRNAs, with 18 DESs (differentially expressed small RNAs). A total of 14,951 DESs of target genes were obtained by miRNA target gene prediction. GO functional classification of differentially expressed miRNAs target genes that were the most strongly affected in the gills of *S. paramamosain* in response to a sudden drop in salinity from 23‰ to 3‰ indicated that 578 genes were associated with cellular processes, 523 with metabolic processes, and 422 with single-organism processes. KEGG pathway analysis of differentially expressed miRNAs target genes showed that most of the identified pathways were associated with metabolism, including 14 pathways related to amino acid metabolism, which plays an important role in osmoregulation. Besides, several pathways were associated with energy metabolism, such as starch and sucrose metabolism (ko00500), glycosaminoglycan degradation (ko00531), and galactose metabolism (ko00052), among others. The above pathways reveal that *S. paramamosain* regulates osmotic pressure and energy balance by regulating target genes to adapt to a sudden change in salinity from 23‰ to 3‰. This article for the first time preliminarily studied the adaptation mechanisms of *S. paramamosain* to a sudden drop in salinity. Furthermore, some novel miRNAs were discovered, which paves the way for further in-depth exploration on salinity adaptation of *S. paramamosain*.

## Additional file

Additional file 1: **Table S1.** Differentially expressed miRNAs. **Table S2.** Gene expression of 18 miRNAs for the comparison of CK and LS groups according to qPCR analysis. **Table S3.** KEGG pathway annotation and classification. (DOCX 60 kb)

## Abbreviations

DESs: Differentially expressed small RNAs
GO: Gene Ontology
KEGG: Kyoto encyclopedia of genes and genomes
RT-qPCR: Quantitative reverse transcription PCR
*S. paramamosain*: *Scylla paramamosain*
sRNAs: Small RNAs
