# Supplementary material for: Identification and characterization of miRNAs in the gills of the mud crab (Scylla paramamosain) in response to a sudden drop in salinity
Source: BMC Genomics. 2018 Aug 14;19:609. doi: 10.1186/s12864-018-4981-6 (PMC6092764; doi:10.1186/s12864-018-4981-6)
Supplement: Supplementary file 1 — Table S1. Differentially expressed miRNAs. Table S2. Gene expression of 18 miRNAs for the comparison of CK and LS groups according to qPCR analysis. Table S3. KEGG pathway annotation and classification. (DOCX 60 kb) [file 12864_2018_4981_MOESM1_ESM.docx]

**Additional file**

**Table S1 Differentially expressed miRNAs**

| **miRNA id** | **Reads (CK)** | **Reads (LS)** | **log_2_Ratio (LS/CK)** | **Regulation** | **P value** | **Q value** |
| --- | --- | --- | --- | --- | --- | --- |
| novel_mir47 | 0 | 43 | 6.542015 | Up | 4.94E-12 | 1.91E-13 |
| novel_mir22 | 0 | 32 | 6.11575 | Up | 2.00E-09 | 6.82E-11 |
| novel_mir40 | 0 | 27 | 5.870637 | Up | 3.44E-08 | 1.12E-09 |
| novel_mir35 | 0 | 23 | 5.639312 | Up | 3.54E-07 | 1.08E-08 |
| novel_mir18 | 0 | 14 | 4.923105 | Up | 8.62E-05 | 2.22E-06 |
| novel_mir19 | 0 | 12 | 4.700712 | Up | 0.000309 | 7.74E-06 |
| novel_mir16 | 31 | 247 | 3.109921 | Up | 3.65E-46 | 2.07E-47 |
| miR-7 | 1157 | 6374 | 2.57756 | Up | 0 | 0 |
| novel_mir26 | 14 | 28 | 1.11575 | Up | 0.014921 | 0.000334 |
| novel_mir53 | 14 | 27 | 1.063282 | Up | 0.021515 | 0.000469 |
| novel_mir5 | 55060 | 25181 | -1.01292 | Down | 0 | 0 |
| novel_mir37 | 37 | 12 | -1.50874 | Down | 0.000684 | 1.66E-05 |
| novel_mir34 | 536 | 172 | -1.52407 | Down | 9.21E-39 | 4.61E-40 |
| novel_mir7 | 67340 | 20469 | -1.60227 | Down | 0 | 0 |
| novel_mir31 | 21 | 0 | -5.27657 | Down | 3.20E-06 | 8.79E-08 |
| novel_mir48 | 23 | 0 | -5.40781 | Down | 1.05E-06 | 2.96E-08 |
| novel_mir24 | 24 | 0 | -5.46921 | Down | 6.00E-07 | 1.76E-08 |
| novel_mir45 | 37 | 0 | -6.0937 | Down | 5.77E-10 | 2.04E-11 |

**Table S2 Gene expression of 18 miRNAs for the comparison of CK and LS groups according to qPCR analysis**

| **ID** | **Ratio** | |
| --- | --- | --- |
|  | **up-regulated (LS / CK)** | **down-regulated (CK / LS)** |
| novel_mir35 | 4.73 | - |
| novel_mir40 | 4.29 | - |
| novel_mir53 | 3.20 | - |
| novel_mir26 | 2.11 | - |
| novel_mir19 | 1.99 | - |
| novel_mir47 | 1.99 | - |
| novel_mir16 | 1.94 | - |
| novel_mir18 | 1.88 | - |
| novel_mir45 | 1.88 | - |
| miR-7 | 1.47 | - |
| novel_mir22 | 1.34 | - |
| novel_mir24 | - | 1.40 |
| novel_mir48 | - | 1.71 |
| novel_mir31 | - | 1.77 |
| novel_mir5 | - | 1.94 |
| novel_mir34 | - | 2.16 |
| novel_mir37 | - | 2.16 |
| novel_mir7 | - | 3.86 |

**Table S3 KEGG pathway annotation and classification**

| **No** | **Pathway** | **DESs target genes with pathway annotation (5019)** | **Qvalue** | **Pathway ID** | **Level 1** |
| --- | --- | --- | --- | --- | --- |
| 1 | Amoebiasis | 455 (5.86%) | 1.52E-15 | ko05146 | Human Diseases |
| 2 | Vibrio cholerae infection | 423 (5.45%) | 1.35E-10 | ko05110 | Human Diseases |
| 3 | Viral carcinogenesis | 118 (1.52%) | 1.25E-02 | ko05203 | Human Diseases |
| 4 | Phosphonate and phosphinate metabolism | 19 (0.24%) | 1.25E-02 | ko00440 | Metabolism |
| 5 | Aminoacyl-tRNA biosynthesis | 52 (0.67%) | 1.25E-02 | ko00970 | Genetic Information Processing |
| 6 | Butanoate metabolism | 24 (0.31%) | 1.25E-02 | ko00650 | Metabolism |
| 7 | Synthesis and degradation of ketone bodies | 14 (0.18%) | 1.47E-02 | ko00072 | Metabolism |
| 8 | IL-17 signaling pathway | 216 (2.78%) | 1.47E-02 | ko04657 | Organismal Systems |
| 9 | Starch and sucrose metabolism | 25 (0.32%) | 1.47E-02 | ko00500 | Metabolism |
| 10 | Hippo signaling pathway -multiple species | 30 (0.39%) | 2.01E-02 | ko04392 | Environmental Information Processing |
| 11 | Apoptosis - fly | 85 (1.09%) | 2.01E-02 | ko04214 | Cellular Processes |
| 12 | Glycosaminoglycan degradation | 22 (0.28%) | 3.52E-02 | ko00531 | Metabolism |
| 13 | Hippo signaling pathway - fly | 115 (1.48%) | 4.73E-02 | ko04391 | Environmental Information Processing |
| 14 | Antifolate resistance | 35 (0.45%) | 5.35E-02 | ko01523 | Human Diseases |
| 15 | One carbon pool by folate | 21 (0.27%) | 1.02E-01 | ko00670 | Metabolism |
| 16 | Galactose metabolism | 33 (0.42%) | 1.45E-01 | ko00052 | Metabolism |
| 17 | Carbohydrate digestion and absorption | 21 (0.27%) | 2.03E-01 | ko04973 | Organismal Systems |
| 18 | Insulin resistance | 73 (0.94%) | 2.03E-01 | ko04931 | Human Diseases |
| 19 | Ovarian steroidogenesis | 37 (0.48%) | 2.03E-01 | ko04913 | Organismal Systems |
| 20 | Hippo signaling pathway | 83 (1.07%) | 2.19E-01 | ko04390 | Environmental Information Processing |
| 21 | Valine, leucine and isoleucine degradation | 37 (0.48%) | 2.19E-01 | ko00280 | Metabolism |
| 22 | AGE-RAGE signaling pathway in diabetic complications | 91 (1.17%) | 3.05E-01 | ko04933 | Human Diseases |
| 23 | Longevity regulating pathway - worm | 55 (0.71%) | 3.05E-01 | ko04212 | Organismal Systems |
| 24 | Aldosterone-regulated sodium reabsorption | 18 (0.23%) | 3.17E-01 | ko04960 | Organismal Systems |
| 25 | Fatty acid degradation | 36 (0.46%) | 3.51E-01 | ko00071 | Metabolism |
| 26 | TNF signaling pathway | 44 (0.57%) | 3.51E-01 | ko04668 | Environmental Information Processing |
| 27 | Steroid hormone biosynthesis | 28 (0.36%) | 3.51E-01 | ko00140 | Metabolism |
| 28 | Glucagon signaling pathway | 88 (1.13%) | 3.51E-01 | ko04922 | Organismal Systems |
| 29 | Toxoplasmosis | 55 (0.71%) | 3.72E-01 | ko05145 | Human Diseases |
| 30 | Valine, leucine and isoleucine biosynthesis | 5 (0.06%) | 3.72E-01 | ko00290 | Metabolism |
| 31 | Bladder cancer | 20 (0.26%) | 3.72E-01 | ko05219 | Human Diseases |
| 32 | Notch signaling pathway | 62 (0.8%) | 3.72E-01 | ko04330 | Environmental Information Processing |
| 33 | RNA degradation | 58 (0.75%) | 4.20E-01 | ko03018 | Genetic Information Processing |
| 34 | Complement and coagulation cascades | 25 (0.32%) | 4.33E-01 | ko04610 | Organismal Systems |
| 35 | Small cell lung cancer | 83 (1.07%) | 4.33E-01 | ko05222 | Human Diseases |
| 36 | Lysosome | 91 (1.17%) | 4.38E-01 | ko04142 | Cellular Processes |
| 37 | Shigellosis | 140 (1.8%) | 4.41E-01 | ko05131 | Human Diseases |
| 38 | Type II diabetes mellitus | 21 (0.27%) | 4.44E-01 | ko04930 | Human Diseases |
| 39 | Renal cell carcinoma | 60 (0.77%) | 4.44E-01 | ko05211 | Human Diseases |
| 40 | Progesterone-mediated oocyte maturation | 48 (0.62%) | 5.06E-01 | ko04914 | Organismal Systems |
| 41 | Mineral absorption | 32 (0.41%) | 5.06E-01 | ko04978 | Organismal Systems |
| 42 | ABC transporters | 32 (0.41%) | 5.06E-01 | ko02010 | Environmental Information Processing |
| 43 | N-Glycan biosynthesis | 42 (0.54%) | 5.36E-01 | ko00510 | Metabolism |
| 44 | Mismatch repair | 15 (0.19%) | 5.45E-01 | ko03430 | Genetic Information Processing |
| 45 | Apoptosis - multiple species | 15 (0.19%) | 5.45E-01 | ko04215 | Cellular Processes |
| 46 | Measles | 50 (0.64%) | 5.50E-01 | ko05162 | Human Diseases |
| 47 | Leukocyte transendothelial migration | 59 (0.76%) | 5.50E-01 | ko04670 | Organismal Systems |
| 48 | RNA transport | 191 (2.46%) | 5.50E-01 | ko03013 | Genetic Information Processing |
| 49 | Jak-STAT signaling pathway | 38 (0.49%) | 5.50E-01 | ko04630 | Environmental Information Processing |
| 50 | Vasopressin-regulated water reabsorption | 25 (0.32%) | 5.50E-01 | ko04962 | Organismal Systems |
| 51 | Pancreatic cancer | 25 (0.32%) | 5.50E-01 | ko05212 | Human Diseases |
| 52 | Toll and Imd signaling pathway | 54 (0.7%) | 5.51E-01 | ko04624 | Organismal Systems |
| 53 | Thyroid hormone synthesis | 58 (0.75%) | 5.52E-01 | ko04918 | Organismal Systems |
| 54 | Platinum drug resistance | 29 (0.37%) | 5.74E-01 | ko01524 | Human Diseases |
| 55 | Folate biosynthesis | 12 (0.15%) | 5.94E-01 | ko00790 | Metabolism |
| 56 | Neurotrophin signaling pathway | 68 (0.88%) | 6.25E-01 | ko04722 | Organismal Systems |
| 57 | ECM-receptor interaction | 128 (1.65%) | 6.25E-01 | ko04512 | Environmental Information Processing |
| 58 | Colorectal cancer | 22 (0.28%) | 6.25E-01 | ko05210 | Human Diseases |
| 59 | Fatty acid metabolism | 33 (0.42%) | 6.25E-01 | ko01212 | Metabolism |
| 60 | Th1 and Th2 cell differentiation | 47 (0.61%) | 6.25E-01 | ko04658 | Organismal Systems |
| 61 | Collecting duct acid secretion | 17 (0.22%) | 6.28E-01 | ko04966 | Organismal Systems |
| 62 | Carbon metabolism | 96 (1.24%) | 6.28E-01 | ko01200 | Metabolism |
| 63 | Selenocompound metabolism | 12 (0.15%) | 6.28E-01 | ko00450 | Metabolism |
| 64 | Oocyte meiosis | 56 (0.72%) | 6.28E-01 | ko04114 | Cellular Processes |
| 65 | Biosynthesis of unsaturated fatty acids | 11 (0.14%) | 6.28E-01 | ko01040 | Metabolism |
| 66 | Hepatitis B | 55 (0.71%) | 6.28E-01 | ko05161 | Human Diseases |
| 67 | Amino sugar and nucleotide sugar metabolism | 54 (0.7%) | 6.28E-01 | ko00520 | Metabolism |
| 68 | Aldosterone synthesis and secretion | 54 (0.7%) | 6.28E-01 | ko04925 | Organismal Systems |
| 69 | Hedgehog signaling pathway | 43 (0.55%) | 6.28E-01 | ko04340 | Environmental Information Processing |
| 70 | Adherens junction | 156 (2.01%) | 6.28E-01 | ko04520 | Cellular Processes |
| 71 | Insect hormone biosynthesis | 17 (0.22%) | 6.34E-01 | ko00981 | Metabolism |
| 72 | Epithelial cell signaling in Helicobacter pylori infection | 42 (0.54%) | 6.34E-01 | ko05120 | Human Diseases |
| 73 | Herpes simplex infection | 196 (2.52%) | 6.57E-01 | ko05168 | Human Diseases |
| 74 | Linoleic acid metabolism | 15 (0.19%) | 6.66E-01 | ko00591 | Metabolism |
| 75 | Cell cycle | 62 (0.8%) | 6.66E-01 | ko04110 | Cellular Processes |
| 76 | beta-Alanine metabolism | 22 (0.28%) | 6.89E-01 | ko00410 | Metabolism |
| 77 | Glutathione metabolism | 38 (0.49%) | 6.89E-01 | ko00480 | Metabolism |
| 78 | Citrate cycle (TCA cycle) | 42 (0.54%) | 6.89E-01 | ko00020 | Metabolism |
| 79 | Histidine metabolism | 13 (0.17%) | 6.89E-01 | ko00340 | Metabolism |
| 80 | Arginine and proline metabolism | 33 (0.42%) | 6.89E-01 | ko00330 | Metabolism |
| 81 | HIF-1 signaling pathway | 68 (0.88%) | 6.93E-01 | ko04066 | Environmental Information Processing |
| 82 | Arachidonic acid metabolism | 25 (0.32%) | 7.10E-01 | ko00590 | Metabolism |
| 83 | Peroxisome | 79 (1.02%) | 7.14E-01 | ko04146 | Cellular Processes |
| 84 | p53 signaling pathway | 27 (0.35%) | 7.68E-01 | ko04115 | Cellular Processes |
| 85 | Dorso-ventral axis formation | 123 (1.58%) | 7.68E-01 | ko04320 | Organismal Systems |
| 86 | Fatty acid elongation | 15 (0.19%) | 7.68E-01 | ko00062 | Metabolism |
| 87 | Melanoma | 12 (0.15%) | 7.69E-01 | ko05218 | Human Diseases |
| 88 | Influenza A | 88 (1.13%) | 7.76E-01 | ko05164 | Human Diseases |
| 89 | Glycosaminoglycan biosynthesis - chondroitin sulfate / dermatan sulfate | 19 (0.24%) | 7.76E-01 | ko00532 | Metabolism |
| 90 | Steroid biosynthesis | 11 (0.14%) | 7.83E-01 | ko00100 | Metabolism |
| 91 | Bile secretion | 59 (0.76%) | 7.83E-01 | ko04976 | Organismal Systems |
| 92 | NF-kappa B signaling pathway | 24 (0.31%) | 7.85E-01 | ko04064 | Environmental Information Processing |
| 93 | Viral myocarditis | 32 (0.41%) | 7.85E-01 | ko05416 | Human Diseases |
| 94 | Fructose and mannose metabolism | 30 (0.39%) | 7.85E-01 | ko00051 | Metabolism |
| 95 | Proximal tubule bicarbonate reclamation | 17 (0.22%) | 7.85E-01 | ko04964 | Organismal Systems |
| 96 | FoxO signaling pathway | 79 (1.02%) | 7.85E-01 | ko04068 | Environmental Information Processing |
| 97 | 2-Oxocarboxylic acid metabolism | 23 (0.3%) | 7.85E-01 | ko01210 | Metabolism |
| 98 | Serotonergic synapse | 42 (0.54%) | 7.85E-01 | ko04726 | Organismal Systems |
| 99 | Chemokine signaling pathway | 90 (1.16%) | 7.85E-01 | ko04062 | Organismal Systems |
| 100 | Biosynthesis of amino acids | 63 (0.81%) | 7.85E-01 | ko01230 | Metabolism |
| 101 | Epstein-Barr virus infection | 226 (2.91%) | 7.93E-01 | ko05169 | Human Diseases |
| 102 | Nicotinate and nicotinamide metabolism | 18 (0.23%) | 7.93E-01 | ko00760 | Metabolism |
| 103 | Inflammatory mediator regulation of TRP channels | 52 (0.67%) | 7.98E-01 | ko04750 | Organismal Systems |
| 104 | DNA replication | 25 (0.32%) | 7.98E-01 | ko03030 | Genetic Information Processing |
| 105 | Ascorbate and aldarate metabolism | 19 (0.24%) | 7.98E-01 | ko00053 | Metabolism |
| 106 | Breast cancer | 62 (0.8%) | 7.98E-01 | ko05224 | Human Diseases |
| 107 | Cocaine addiction | 21 (0.27%) | 7.98E-01 | ko05030 | Human Diseases |
| 108 | Caffeine metabolism | 7 (0.09%) | 8.15E-01 | ko00232 | Metabolism |
| 109 | Prostate cancer | 47 (0.61%) | 8.15E-01 | ko05215 | Human Diseases |
| 110 | Chronic myeloid leukemia | 31 (0.4%) | 8.15E-01 | ko05220 | Human Diseases |
| 111 | Intestinal immune network for IgA production | 3 (0.04%) | 8.15E-01 | ko04672 | Organismal Systems |
| 112 | Endometrial cancer | 21 (0.27%) | 8.23E-01 | ko05213 | Human Diseases |
| 113 | Cholinergic synapse | 49 (0.63%) | 8.23E-01 | ko04725 | Organismal Systems |
| 114 | Rap1 signaling pathway | 116 (1.49%) | 8.23E-01 | ko04015 | Environmental Information Processing |
| 115 | Phagosome | 87 (1.12%) | 8.23E-01 | ko04145 | Cellular Processes |
| 116 | Pyrimidine metabolism | 184 (2.37%) | 8.23E-01 | ko00240 | Metabolism |
| 117 | Tuberculosis | 66 (0.85%) | 8.23E-01 | ko05152 | Human Diseases |
| 118 | Inflammatory bowel disease (IBD) | 8 (0.1%) | 8.23E-01 | ko05321 | Human Diseases |
| 119 | Thyroid hormone signaling pathway | 183 (2.36%) | 8.23E-01 | ko04919 | Organismal Systems |
| 120 | Regulation of lipolysis in adipocytes | 34 (0.44%) | 8.23E-01 | ko04923 | Organismal Systems |
| 121 | mRNA surveillance pathway | 115 (1.48%) | 8.28E-01 | ko03015 | Genetic Information Processing |
| 122 | Leishmaniasis | 19 (0.24%) | 8.31E-01 | ko05140 | Human Diseases |
| 123 | Morphine addiction | 46 (0.59%) | 8.31E-01 | ko05032 | Human Diseases |
| 124 | HTLV-I infection | 115 (1.48%) | 8.31E-01 | ko05166 | Human Diseases |
| 125 | Sulfur metabolism | 7 (0.09%) | 8.31E-01 | ko00920 | Metabolism |
| 126 | Tight junction | 193 (2.48%) | 8.31E-01 | ko04530 | Cellular Processes |
| 127 | Pyruvate metabolism | 35 (0.45%) | 8.50E-01 | ko00620 | Metabolism |
| 128 | Salmonella infection | 142 (1.83%) | 8.61E-01 | ko05132 | Human Diseases |
| 129 | Malaria | 12 (0.15%) | 8.61E-01 | ko05144 | Human Diseases |
| 130 | Primary bile acid biosynthesis | 8 (0.1%) | 8.63E-01 | ko00120 | Metabolism |
| 131 | Ribosome biogenesis in eukaryotes | 63 (0.81%) | 8.78E-01 | ko03008 | Genetic Information Processing |
| 132 | Prion diseases | 36 (0.46%) | 8.78E-01 | ko05020 | Human Diseases |
| 133 | Fat digestion and absorption | 23 (0.3%) | 8.78E-01 | ko04975 | Organismal Systems |
| 134 | D-Glutamine and D-glutamate metabolism | 3 (0.04%) | 8.78E-01 | ko00471 | Metabolism |
| 135 | Tryptophan metabolism | 21 (0.27%) | 8.82E-01 | ko00380 | Metabolism |
| 136 | Renin-angiotensin system | 14 (0.18%) | 8.82E-01 | ko04614 | Organismal Systems |
| 137 | Nucleotide excision repair | 35 (0.45%) | 8.82E-01 | ko03420 | Genetic Information Processing |
| 138 | Focal adhesion | 219 (2.82%) | 8.82E-01 | ko04510 | Cellular Processes |
| 139 | SNARE interactions in vesicular transport | 12 (0.15%) | 8.85E-01 | ko04130 | Genetic Information Processing |
| 140 | ErbB signaling pathway | 34 (0.44%) | 8.94E-01 | ko04012 | Environmental Information Processing |
| 141 | Central carbon metabolism in cancer | 37 (0.48%) | 8.94E-01 | ko05230 | Human Diseases |
| 142 | Nitrogen metabolism | 6 (0.08%) | 9.08E-01 | ko00910 | Metabolism |
| 143 | Neomycin, kanamycin and gentamicin biosynthesis | 2 (0.03%) | 9.08E-01 | ko00524 | Metabolism |
| 144 | alpha-Linolenic acid metabolism | 9 (0.12%) | 9.16E-01 | ko00592 | Metabolism |
| 145 | Circadian rhythm | 20 (0.26%) | 9.16E-01 | ko04710 | Organismal Systems |
| 146 | Protein digestion and absorption | 123 (1.58%) | 9.16E-01 | ko04974 | Organismal Systems |
| 147 | Chemical carcinogenesis | 26 (0.33%) | 9.16E-01 | ko05204 | Human Diseases |
| 148 | Calcium signaling pathway | 147 (1.89%) | 9.17E-01 | ko04020 | Environmental Information Processing |
| 149 | Mannose type O-glycan biosynthesis | 18 (0.23%) | 9.17E-01 | ko00515 | Metabolism |
| 150 | Arginine biosynthesis | 10 (0.13%) | 9.21E-01 | ko00220 | Metabolism |
| 151 | Staphylococcus aureus infection | 5 (0.06%) | 9.21E-01 | ko05150 | Human Diseases |
| 152 | Circadian rhythm - fly | 22 (0.28%) | 9.21E-01 | ko04711 | Organismal Systems |
| 153 | Fanconi anemia pathway | 32 (0.41%) | 9.28E-01 | ko03460 | Genetic Information Processing |
| 154 | Endocrine resistance | 125 (1.61%) | 9.30E-01 | ko01522 | Human Diseases |
| 155 | Vitamin B6 metabolism | 3 (0.04%) | 9.30E-01 | ko00750 | Metabolism |
| 156 | Sphingolipid metabolism | 27 (0.35%) | 9.30E-01 | ko00600 | Metabolism |
| 157 | Monobactam biosynthesis | 1 (0.01%) | 9.45E-01 | ko00261 | Metabolism |
| 158 | Long-term potentiation | 42 (0.54%) | 9.45E-01 | ko04720 | Organismal Systems |
| 159 | Protein export | 13 (0.17%) | 9.48E-01 | ko03060 | Genetic Information Processing |
| 160 | Propanoate metabolism | 17 (0.22%) | 9.57E-01 | ko00640 | Metabolism |
| 161 | Bacterial invasion of epithelial cells | 132 (1.7%) | 9.61E-01 | ko05100 | Human Diseases |
| 162 | Antigen processing and presentation | 29 (0.37%) | 9.70E-01 | ko04612 | Organismal Systems |
| 163 | Retrograde endocannabinoid signaling | 47 (0.61%) | 9.70E-01 | ko04723 | Organismal Systems |
| 164 | Drug metabolism - cytochrome P450 | 16 (0.21%) | 9.77E-01 | ko00982 | Metabolism |
| 165 | Biotin metabolism | 2 (0.03%) | 9.80E-01 | ko00780 | Metabolism |
| 166 | TGF-beta signaling pathway | 32 (0.41%) | 9.80E-01 | ko04350 | Environmental Information Processing |
| 167 | Glioma | 25 (0.32%) | 9.80E-01 | ko05214 | Human Diseases |
| 168 | Other glycan degradation | 14 (0.18%) | 9.80E-01 | ko00511 | Metabolism |
| 169 | Basal transcription factors | 31 (0.4%) | 9.80E-01 | ko03022 | Genetic Information Processing |
| 170 | Osteoclast differentiation | 35 (0.45%) | 9.80E-01 | ko04380 | Organismal Systems |
| 171 | Lipoic acid metabolism | 3 (0.04%) | 9.80E-01 | ko00785 | Metabolism |
| 172 | Systemic lupus erythematosus | 12 (0.15%) | 9.80E-01 | ko05322 | Human Diseases |
| 173 | Glycosaminoglycan biosynthesis - keratan sulfate | 8 (0.1%) | 9.80E-01 | ko00533 | Metabolism |
| 174 | Adipocytokine signaling pathway | 41 (0.53%) | 9.80E-01 | ko04920 | Organismal Systems |
| 175 | RIG-I-like receptor signaling pathway | 17 (0.22%) | 9.80E-01 | ko04622 | Organismal Systems |
| 176 | Chagas disease (American trypanosomiasis) | 34 (0.44%) | 9.80E-01 | ko05142 | Human Diseases |
| 177 | Hepatitis C | 42 (0.54%) | 9.80E-01 | ko05160 | Human Diseases |
| 178 | Acute myeloid leukemia | 22 (0.28%) | 9.80E-01 | ko05221 | Human Diseases |
| 179 | Glycerolipid metabolism | 45 (0.58%) | 9.92E-01 | ko00561 | Metabolism |
| 180 | Wnt signaling pathway | 77 (0.99%) | 9.92E-01 | ko04310 | Environmental Information Processing |
| 181 | Cardiac muscle contraction | 27 (0.35%) | 1.00E+00 | ko04260 | Organismal Systems |
| 182 | Glycosylphosphatidylinositol (GPI)-anchor biosynthesis | 10 (0.13%) | 1.00E+00 | ko00563 | Metabolism |
| 183 | Toll-like receptor signaling pathway | 26 (0.33%) | 1.00E+00 | ko04620 | Organismal Systems |
| 184 | Purine metabolism | 258 (3.32%) | 1.00E+00 | ko00230 | Metabolism |
| 185 | PPAR signaling pathway | 36 (0.46%) | 1.00E+00 | ko03320 | Organismal Systems |
| 186 | Vitamin digestion and absorption | 18 (0.23%) | 1.00E+00 | ko04977 | Organismal Systems |
| 187 | Th17 cell differentiation | 30 (0.39%) | 1.00E+00 | ko04659 | Organismal Systems |
| 188 | Porphyrin and chlorophyll metabolism | 15 (0.19%) | 1.00E+00 | ko00860 | Metabolism |
| 189 | Mucin type O-glycan biosynthesis | 8 (0.1%) | 1.00E+00 | ko00512 | Metabolism |
| 190 | RNA polymerase | 139 (1.79%) | 1.00E+00 | ko03020 | Genetic Information Processing |
| 191 | Oxidative phosphorylation | 53 (0.68%) | 1.00E+00 | ko00190 | Metabolism |
| 192 | Glyoxylate and dicarboxylate metabolism | 19 (0.24%) | 1.00E+00 | ko00630 | Metabolism |
| 193 | Glycosaminoglycan biosynthesis - heparan sulfate / heparin | 13 (0.17%) | 1.00E+00 | ko00534 | Metabolism |
| 194 | Pancreatic secretion | 96 (1.24%) | 1.00E+00 | ko04972 | Organismal Systems |
| 195 | Gastric acid secretion | 43 (0.55%) | 1.00E+00 | ko04971 | Organismal Systems |
| 196 | Pathogenic Escherichia coli infection | 74 (0.95%) | 1.00E+00 | ko05130 | Human Diseases |
| 197 | Fatty acid biosynthesis | 8 (0.1%) | 1.00E+00 | ko00061 | Metabolism |
| 198 | Other types of O-glycan biosynthesis | 13 (0.17%) | 1.00E+00 | ko00514 | Metabolism |
| 199 | Nicotine addiction | 16 (0.21%) | 1.00E+00 | ko05033 | Human Diseases |
| 200 | Alcoholism | 43 (0.55%) | 1.00E+00 | ko05034 | Human Diseases |
| 201 | Drug metabolism - other enzymes | 37 (0.48%) | 1.00E+00 | ko00983 | Metabolism |
| 202 | Alzheimer's disease | 93 (1.2%) | 1.00E+00 | ko05010 | Human Diseases |
| 203 | Fc epsilon RI signaling pathway | 24 (0.31%) | 1.00E+00 | ko04664 | Organismal Systems |
| 204 | Pentose and glucuronate interconversions | 24 (0.31%) | 1.00E+00 | ko00040 | Metabolism |
| 205 | Circadian entrainment | 46 (0.59%) | 1.00E+00 | ko04713 | Organismal Systems |
| 206 | Metabolism of xenobiotics by cytochrome P450 | 16 (0.21%) | 1.00E+00 | ko00980 | Metabolism |
| 207 | Pentose phosphate pathway | 17 (0.22%) | 1.00E+00 | ko00030 | Metabolism |
| 208 | Melanogenesis | 45 (0.58%) | 1.00E+00 | ko04916 | Organismal Systems |
| 209 | Non-alcoholic fatty liver disease (NAFLD) | 59 (0.76%) | 1.00E+00 | ko04932 | Human Diseases |
| 210 | NOD-like receptor signaling pathway | 54 (0.7%) | 1.00E+00 | ko04621 | Organismal Systems |
| 211 | Arrhythmogenic right ventricular cardiomyopathy (ARVC) | 34 (0.44%) | 1.00E+00 | ko05412 | Human Diseases |
| 212 | Estrogen signaling pathway | 58 (0.75%) | 1.00E+00 | ko04915 | Organismal Systems |
| 213 | Alanine, aspartate and glutamate metabolism | 15 (0.19%) | 1.00E+00 | ko00250 | Metabolism |
| 214 | Prolactin signaling pathway | 24 (0.31%) | 1.00E+00 | ko04917 | Organismal Systems |
| 215 | Ubiquitin mediated proteolysis | 146 (1.88%) | 1.00E+00 | ko04120 | Genetic Information Processing |
| 216 | Hedgehog signaling pathway - fly | 30 (0.39%) | 1.00E+00 | ko04341 | Environmental Information Processing |
| 217 | Apelin signaling pathway | 66 (0.85%) | 1.00E+00 | ko04371 | Environmental Information Processing |
| 218 | Riboflavin metabolism | 3 (0.04%) | 1.00E+00 | ko00740 | Metabolism |
| 219 | Gap junction | 57 (0.73%) | 1.00E+00 | ko04540 | Cellular Processes |
| 220 | Glycolysis / Gluconeogenesis | 54 (0.7%) | 1.00E+00 | ko00010 | Metabolism |
| 221 | Glycine, serine and threonine metabolism | 45 (0.58%) | 1.00E+00 | ko00260 | Metabolism |
| 222 | Terpenoid backbone biosynthesis | 9 (0.12%) | 1.00E+00 | ko00900 | Metabolism |
| 223 | Phototransduction - fly | 23 (0.3%) | 1.00E+00 | ko04745 | Organismal Systems |
| 224 | Amphetamine addiction | 24 (0.31%) | 1.00E+00 | ko05031 | Human Diseases |
| 225 | Taste transduction | 18 (0.23%) | 1.00E+00 | ko04742 | Organismal Systems |
| 226 | Pertussis | 17 (0.22%) | 1.00E+00 | ko05133 | Human Diseases |
| 227 | Regulation of actin cytoskeleton | 264 (3.4%) | 1.00E+00 | ko04810 | Cellular Processes |
| 228 | Pantothenate and CoA biosynthesis | 7 (0.09%) | 1.00E+00 | ko00770 | Metabolism |
| 229 | T cell receptor signaling pathway | 33 (0.42%) | 1.00E+00 | ko04660 | Organismal Systems |
| 230 | Protein processing in endoplasmic reticulum | 115 (1.48%) | 1.00E+00 | ko04141 | Genetic Information Processing |
| 231 | Cytosolic DNA-sensing pathway | 48 (0.62%) | 1.00E+00 | ko04623 | Organismal Systems |
| 232 | Ubiquinone and other terpenoid-quinone biosynthesis | 12 (0.15%) | 1.00E+00 | ko00130 | Metabolism |
| 233 | GABAergic synapse | 40 (0.51%) | 1.00E+00 | ko04727 | Organismal Systems |
| 234 | Dopaminergic synapse | 54 (0.7%) | 1.00E+00 | ko04728 | Organismal Systems |
| 235 | Axon guidance | 103 (1.33%) | 1.00E+00 | ko04360 | Organismal Systems |
| 236 | Autoimmune thyroid disease | 3 (0.04%) | 1.00E+00 | ko05320 | Human Diseases |
| 237 | Huntington's disease | 196 (2.52%) | 1.00E+00 | ko05016 | Human Diseases |
| 238 | Non-homologous end-joining | 4 (0.05%) | 1.00E+00 | ko03450 | Genetic Information Processing |
| 239 | Renin secretion | 38 (0.49%) | 1.00E+00 | ko04924 | Organismal Systems |
| 240 | Primary immunodeficiency | 3 (0.04%) | 1.00E+00 | ko05340 | Human Diseases |
| 241 | Long-term depression | 25 (0.32%) | 1.00E+00 | ko04730 | Organismal Systems |
| 242 | Choline metabolism in cancer | 195 (2.51%) | 1.00E+00 | ko05231 | Human Diseases |
| 243 | Homologous recombination | 18 (0.23%) | 1.00E+00 | ko03440 | Genetic Information Processing |
| 244 | Legionellosis | 30 (0.39%) | 1.00E+00 | ko05134 | Human Diseases |
| 245 | Lysine degradation | 125 (1.61%) | 1.00E+00 | ko00310 | Metabolism |
| 246 | Natural killer cell mediated cytotoxicity | 26 (0.33%) | 1.00E+00 | ko04650 | Organismal Systems |
| 247 | Oxytocin signaling pathway | 71 (0.91%) | 1.00E+00 | ko04921 | Organismal Systems |
| 248 | Thiamine metabolism | 5 (0.06%) | 1.00E+00 | ko00730 | Metabolism |
| 249 | Non-small cell lung cancer | 21 (0.27%) | 1.00E+00 | ko05223 | Human Diseases |
| 250 | Cysteine and methionine metabolism | 30 (0.39%) | 1.00E+00 | ko00270 | Metabolism |
| 251 | Apoptosis | 70 (0.9%) | 1.00E+00 | ko04210 | Cellular Processes |
| 252 | Tyrosine metabolism | 12 (0.15%) | 1.00E+00 | ko00350 | Metabolism |
| 253 | VEGF signaling pathway | 32 (0.41%) | 1.00E+00 | ko04370 | Environmental Information Processing |
| 254 | Hypertrophic cardiomyopathy (HCM) | 66 (0.85%) | 1.00E+00 | ko05410 | Human Diseases |
| 255 | Proteasome | 20 (0.26%) | 1.00E+00 | ko03050 | Genetic Information Processing |
| 256 | MAPK signaling pathway - fly | 102 (1.31%) | 1.00E+00 | ko04013 | Environmental Information Processing |
| 257 | Type I diabetes mellitus | 4 (0.05%) | 1.00E+00 | ko04940 | Human Diseases |
| 258 | Dilated cardiomyopathy | 74 (0.95%) | 1.00E+00 | ko05414 | Human Diseases |
| 259 | Rheumatoid arthritis | 21 (0.27%) | 1.00E+00 | ko05323 | Human Diseases |
| 260 | Pathways in cancer | 255 (3.28%) | 1.00E+00 | ko05200 | Human Diseases |
| 261 | Sulfur relay system | 3 (0.04%) | 1.00E+00 | ko04122 | Genetic Information Processing |
| 262 | Amyotrophic lateral sclerosis (ALS) | 37 (0.48%) | 1.00E+00 | ko05014 | Human Diseases |
| 263 | Glycosphingolipid biosynthesis - globo and isoglobo series | 18 (0.23%) | 1.00E+00 | ko00603 | Metabolism |
| 264 | Thyroid cancer | 14 (0.18%) | 1.00E+00 | ko05216 | Human Diseases |
| 265 | Glycosphingolipid biosynthesis - ganglio series | 17 (0.22%) | 1.00E+00 | ko00604 | Metabolism |
| 266 | Fluid shear stress and atherosclerosis | 78 (1%) | 1.00E+00 | ko05418 | Human Diseases |
| 267 | Insulin signaling pathway | 136 (1.75%) | 1.00E+00 | ko04910 | Organismal Systems |
| 268 | Insulin secretion | 59 (0.76%) | 1.00E+00 | ko04911 | Organismal Systems |
| 269 | Metabolic pathways | 1009 (12.99%) | 1.00E+00 | ko01100 | Metabolism |
| 270 | Lysine biosynthesis | 1 (0.01%) | 1.00E+00 | ko00300 | Metabolism |
| 271 | Adrenergic signaling in cardiomyocytes | 76 (0.98%) | 1.00E+00 | ko04261 | Organismal Systems |
| 272 | Signaling pathways regulating pluripotency of stem cells | 39 (0.5%) | 1.00E+00 | ko04550 | Cellular Processes |
| 273 | Parkinson's disease | 40 (0.51%) | 1.00E+00 | ko05012 | Human Diseases |
| 274 | Phenylalanine metabolism | 7 (0.09%) | 1.00E+00 | ko00360 | Metabolism |
| 275 | Cytokine-cytokine receptor interaction | 19 (0.24%) | 1.00E+00 | ko04060 | Environmental Information Processing |
| 276 | Glycosphingolipid biosynthesis - lacto and neolacto series | 17 (0.22%) | 1.00E+00 | ko00601 | Metabolism |
| 277 | Retinol metabolism | 62 (0.8%) | 1.00E+00 | ko00830 | Metabolism |
| 278 | Base excision repair | 12 (0.15%) | 1.00E+00 | ko03410 | Genetic Information Processing |
| 279 | Salivary secretion | 79 (1.02%) | 1.00E+00 | ko04970 | Organismal Systems |
| 280 | MicroRNAs in cancer | 176 (2.27%) | 1.00E+00 | ko05206 | Human Diseases |
| 281 | Phenylalanine, tyrosine and tryptophan biosynthesis | 2 (0.03%) | 1.00E+00 | ko00400 | Metabolism |
| 282 | Basal cell carcinoma | 15 (0.19%) | 1.00E+00 | ko05217 | Human Diseases |
| 283 | Glycerophospholipid metabolism | 93 (1.2%) | 1.00E+00 | ko00564 | Metabolism |
| 284 | Endocrine and other factor-regulated calcium reabsorption | 26 (0.33%) | 1.00E+00 | ko04961 | Organismal Systems |
| 285 | Synaptic vesicle cycle | 26 (0.33%) | 1.00E+00 | ko04721 | Organismal Systems |
| 286 | Phosphatidylinositol signaling system | 86 (1.11%) | 1.00E+00 | ko04070 | Environmental Information Processing |
| 287 | African trypanosomiasis | 3 (0.04%) | 1.00E+00 | ko05143 | Human Diseases |
| 288 | AMPK signaling pathway | 98 (1.26%) | 1.00E+00 | ko04152 | Environmental Information Processing |
| 289 | Endocytosis | 371 (4.78%) | 1.00E+00 | ko04144 | Cellular Processes |
| 290 | MAPK signaling pathway | 169 (2.18%) | 1.00E+00 | ko04010 | Environmental Information Processing |
| 291 | Longevity regulating pathway | 98 (1.26%) | 1.00E+00 | ko04211 | Organismal Systems |
| 292 | Vascular smooth muscle contraction | 103 (1.33%) | 1.00E+00 | ko04270 | Organismal Systems |
| 293 | Autophagy - animal | 112 (1.44%) | 1.00E+00 | ko04140 | Cellular Processes |
| 294 | mTOR signaling pathway | 117 (1.51%) | 1.00E+00 | ko04150 | Environmental Information Processing |
| 295 | Maturity onset diabetes of the young | 2 (0.03%) | 1.00E+00 | ko04950 | Human Diseases |
| 296 | Ribosome | 73 (0.94%) | 1.00E+00 | ko03010 | Genetic Information Processing |
| 297 | Longevity regulating pathway - multiple species | 76 (0.98%) | 1.00E+00 | ko04213 | Organismal Systems |
| 298 | Platelet activation | 116 (1.49%) | 1.00E+00 | ko04611 | Organismal Systems |
| 299 | Inositol phosphate metabolism | 79 (1.02%) | 1.00E+00 | ko00562 | Metabolism |
| 300 | Sphingolipid signaling pathway | 68 (0.88%) | 1.00E+00 | ko04071 | Environmental Information Processing |
| 301 | cGMP-PKG signaling pathway | 140 (1.8%) | 1.00E+00 | ko04022 | Environmental Information Processing |
| 302 | Ether lipid metabolism | 44 (0.57%) | 1.00E+00 | ko00565 | Metabolism |
| 303 | GnRH signaling pathway | 71 (0.91%) | 1.00E+00 | ko04912 | Organismal Systems |
| 304 | Transcriptional misregulation in cancer | 171 (2.2%) | 1.00E+00 | ko05202 | Human Diseases |
| 305 | Ras signaling pathway | 166 (2.14%) | 1.00E+00 | ko04014 | Environmental Information Processing |
| 306 | Spliceosome | 191 (2.46%) | 1.00E+00 | ko03040 | Genetic Information Processing |
| 307 | PI3K-Akt signaling pathway | 287 (3.69%) | 1.00E+00 | ko04151 | Environmental Information Processing |
| 308 | Phospholipase D signaling pathway | 161 (2.07%) | 1.00E+00 | ko04072 | Environmental Information Processing |
| 309 | Phototransduction | 17 (0.22%) | 1.00E+00 | ko04744 | Organismal Systems |
| 310 | Fc gamma R-mediated phagocytosis | 168 (2.16%) | 1.00E+00 | ko04666 | Organismal Systems |
| 311 | cAMP signaling pathway | 141 (1.82%) | 1.00E+00 | ko04024 | Environmental Information Processing |
| 312 | Hematopoietic cell lineage | 118 (1.52%) | 1.00E+00 | ko04640 | Organismal Systems |
| 313 | Glutamatergic synapse | 131 (1.69%) | 1.00E+00 | ko04724 | Organismal Systems |
| 314 | Cell adhesion molecules (CAMs) | 141 (1.82%) | 1.00E+00 | ko04514 | Environmental Information Processing |
| 315 | Olfactory transduction | 39 (0.5%) | 1.00E+00 | ko04740 | Organismal Systems |
| 316 | Neuroactive ligand-receptor interaction | 119 (1.53%) | 1.00E+00 | ko04080 | Environmental Information Processing |
| 317 | EGFR tyrosine kinase inhibitor resistance | 77 (0.99%) | 1.00E+00 | ko01521 | Human Diseases |
| 318 | B cell receptor signaling pathway | 130 (1.67%) | 1.00E+00 | ko04662 | Organismal Systems |
| 319 | Proteoglycans in cancer | 177 (2.28%) | 1.00E+00 | ko05205 | Human Diseases |
